# Brain organoids and genome editing: A new era in understanding human brain development and disorders

**DOI:** 10.4103/NRR.NRR-D-24-01546

**Published:** 2025-07-05

**Authors:** Min Zhou, Yuanqing Cao, Ke Yue, Wenyu Wu, Yutong Xie, Daiyu Hu, Jingjing Zhao, Fang Xu, Jianrong Guo, Zhenzhou Li, Huan Wang, Zhengliang Gao

**Affiliations:** 1 Clinical Research Center for Mental Health, Mental Health Center Affiliated to School of Medicine (Shanghai Hongkou Mental Health Center), Shanghai University, Shanghai, China; 2 Department of Anesthesiology and Perioperative Medicine, General Hospital of Ningxia Medical University, Yinchuan, Ningxia Hui Autonomous Region, China; 3 Department of Anesthesiology, Gongli Hospital of Shanghai Pudong New Area, Shanghai, China; 4 Fundamental Research Center, Shanghai Yangzhi Rehabilitation Hospital (Shanghai Sunshine Rehabilitation Center), School of Medicine, Tongji University, Shanghai, China; 5 School of Basic Medicine, Medical Genetics and Cell Biology, Ningxia Medical University, Yinchuan, Ningxia Hui Autonomous Region, China; 6 China-Japan Friendship Medical Research Institute, School of Medicine, Shanghai University, Shanghai, China

**Keywords:** brain disorder, brain organoid, CRISPR/Cas9, disease modeling, drug screening, genome editing, human brain development, lineage tracing, organoid modeling, stem cell differentiation

## Abstract

Brain organoids are artificial neural tissues derived *in vitro*, containing a variety of cell types, as well as structural and/or functional brain regions. They can partially mimic brain physiological activities and diseased processes. Owing to their operability and sample accessibility, brain organoids serve as a bridge between *in vitro* monolayer cell culture models and *in vivo* animal models. An increasing number of induction protocols for brain organoids have been developed over the preceding decade. A key future research direction will focus on ensuring the complexity and quality of brain organoids. The integration of powerful technologies, such as the CRISPR/Cas9 genome editing and lineage tracing systems, shall precipitate practical and broad applications of brain organoids. In this review, we discuss the generation and application of brain organoids, as well as their integration with genome editing technologies, in the study of neural development, disease modeling, and mechanistic investigations. The innovative combination of these two technologies may offer a fresh perspective for exploring the fundamental aspects of the human nervous system and related diseases.

## Introduction

Understanding human brain development and disorders is one of the most compelling challenges in life sciences. The primary models currently used for neuroscience studies are rodents and two-dimensional (2D) cell cultures due to the limited availability of fresh human brain tissue. However, these models are characterized by significant limitations and challenges in drug screening, as well as preclinical and clinical studies. Consequently, there is an urgent need for functional, practical, and personalized models of human brain development and diseases. The advent of pluripotent stem cells (PSCs) and their ability to differentiate *in vitro* has paved the way for the construction of three-dimensional (3D) models of developing neural tissue. *In vitro*, PSCs can proliferate and, upon exquisite induction, differentiate into specific cell types and form 3D tissue mimics (e.g., aggregates of brain cells). These artificial brain tissues, derived *in vitro* and referred to as brain organoids, consist of various cell types, as well as structural and functional brain regions. Brain organoids can form cellular arrangements resembling human brain structures, including the human-specific outer subventricular zone (OSVZ) (Kadoshima et al., 2013; Qian et al., 2016). Comparisons between brain organoids and human brain tissues during fetal development revealed comparable gene expression programs (Camp et al., 2015). Various brain organoid induction protocols have been established to model human brain development and pathogenesis, such as microcephaly (Fair et al., 2023; Ijezie et al., 2023) and Zika virus infection (Qian et al., 2016; Krenn et al., 2021; Li et al., 2022; Slonchak et al., 2022). Nonetheless, a number of significant challenges remain, including uncontrolled growth, size limitations, and the lack of physiological anatomy (Lee et al., 2017; Andrews and Kriegstein, 2022). Moreover, current protocols primarily simulate early development and must be further evaluated for adult brain physiology and pathology modeling.

Genome editing technology, as a major technological breakthrough, has been rapidly adopted across fields. It offers new exciting promise with broad utilities in basic research and clinical application (Doudna, 2020; Bhattacharjee et al., 2022). In particular, the clustered regularly interspaced short palindromic repeat (CRISPR)/CRISPR-associated nuclease 9 (Cas9) editing system has rapidly become the predominant genome editing method due to its simplicity and accessibility. Scientists have been actively trying to combine genome editing with brain organoid culture to dissect key human neurodevelopmental and pathological questions otherwise impossible or challenging to address (Chen et al., 2021a). To date, little effort has been dedicated to addressing the combination of the two powerful technologies. In this review, we summarize recent advancements in brain organoid research and the application of genome editing technologies in this context. We also assess their potential applications in neural development, disease modeling, and regenerative therapies, while highlighting the obstacles and challenges faced in this field.

## Search Strategy

To obtain potentially relevant literature, we used the PubMed database and various combinations of the following keywords: brain organoid, genome editing, CRISPR/Cas9, neural development, and neural diseases. We screened the literature based on the topic of this article, assessed relevance through the title and abstract, and subsequently reviewed the eligible articles in detail before summarizing the information they contain. All selected references were published in English; except for seminal historical articles in the field, most references were published between 2010 and 2024, accounting for > 90% of the references.

## Generating Three-Dimensional Human Brain Organoids

### Historical and developmental perspectives of brain organoids

**Figure 1:**
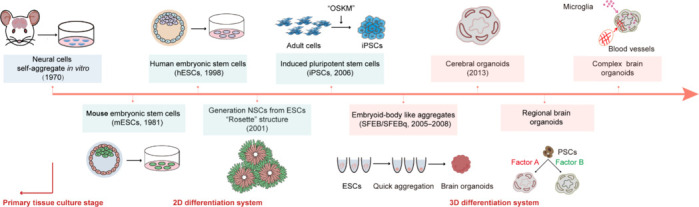
Timeline of studies in neuroscience *in vitro*. Summary of the development history and breakthroughs in the field of brain organoids. During the early stages of tissue culture, researchers discovered that isolated neural cells could be cultured *in vitro* and were capable of self-aggregation. With the advent of embryonic stem cells and induced pluripotent stem cells, these cells can be differentiated into neural stem cells *in vitro*, which exhibit distinct neural tube-like structures known as rosettes. Advances in tissue culture techniques and 2D cell culture have laid the groundwork for brain organoid technology, and the evolution of organoid methods has heralded a new era in neural culture. 2D: Two-dimensional; 3D: three-dimensional; ESCs: embryonic stem cells; NSCs: neural stem cells; OSKM: Oct3/4, Sox2, c-Myc, and Klf4; PSCs: pluripotent stem cells; SFEB/SFEBq: floating embryoid body-like aggregate culture/serum-free floating culture of EB-like aggregates with quick reaggregation.

It has been documented long before the modern organoid era that mammalian brain tissue could be cultured *in vitro* (**Figure 1**; DeLong, 1990). Samples dissociated from tissues into single cells tend to aggregate in culture and exhibit the same structure as the original tissue. Following the primary tissue culture phase, neural research advanced to the stem cell era. Evans and Kaufman (1981) isolated cells from the inner cell mass of late-stage mouse blastocysts, which, when transplanted into mice, formed teratomas. These cells were formally designated as mouse embryonic stem cells (ESCs). In 1998, Thomson et al. isolated human ESC (hESC) lines. Subsequently, Zhang et al. (2001) derived neural progenitor cells (NPCs) from hESCs using a 2D differentiation system. In the presence of fibroblast growth factor 2 (FGF2), NPCs formed neural tube-like structures called rosettes. These progresses initiated a surge in the study of neural development using 2D models. Takahashi and Yamanaka (2006) achieved a major breakthrough by generating induced pluripotent stem cells (iPSCs) through ectopic overexpression of four transcription factors, namely octamer-binding protein 3/4 (Oct3/4), SRY-box transcription factor 2 (Sox2), Krüppel-like factor 4 (Klf4), and c-Myc. Ever since, the advent of iPSCs has markedly accelerated and continuously advanced the field of neural disease research, as it provides unique and powerful approaches to model and study diseases with patient-derived cells. The iPSCs could even retain epigenetic information from patients, such as DNA methylation, acetylation, and specific gene expression, which are crucial for personalized research (Doi et al., 2009; Kim et al., 2010; Noguchi et al., 2018; Scesa et al., 2021). The hESCs and human iPSCs (hiPSCs), collectively termed human PSCs, are central to studying human neural development and diseases.

The human nervous system is extraordinarily complex. Numerous interactions of varied nature occur between cells, extracellular matrix, and soluble molecules. Neurulation and development occur as cells proliferate, differentiate, and move into distinct locations in a highly spatiotemporally orchestrated manner, adopting different fates of diverse functions, responsive to key secreted signaling molecules called morphogens (Gurdon and Bourillot, 2001). Varying concentrations of these molecules, termed morphogen gradients, are key spatiotemporal cues driving brain development along the anterior–posterior (AP) and dorsal–ventral (DV) axes (Cadwell et al., 2019). The sonic hedgehog (SHH) pathway regulates the formation of the DV axis (Hall et al., 2024), while the wingless-related integration site (WNT) pathway governs the formation of the AP axis (Kühl et al., 2000; Mulligan and Cheyette, 2012). Bone morphogenetic protein (BMP), a member of the transforming growth factor-beta (TGF-β) superfamily, displays a pattern of high concentration dorsally and low concentration ventrally, playing important roles in ectodermal fate regulation and DV axis formation (Bier and De Robertis, 2015). However, in conventional 2D monolayer culture, cells without 3D geometry and morphogen gradients tend to lose intrinsic properties, hindering development and disease research.

To address these limitations, 3D culture techniques emerged. In 2005, Yoshiki Sasai’s group developed a serum-free, floating culture method for embryoid body-like aggregates, and in 2008, achieved self-organized formation of polarized cortical tissues through improved serum-free floating culture of EB-like aggregates with quick reaggregation (SFEBq) method (Watanabe et al., 2005; Eiraku et al., 2008). These studies precipitated the field of brain organoids. In 2011, Eiraku et al. (2011) (also from Sasai’s group), optimized the SFEBq by adding Matrigel to provide rigid support and became the first to induce the formation of optic cup structures from PSCs through self-organization and differentiation. Thereafter, in a major 2013 breakthrough, Lancaster et al. reported the self-organized formation of 3D structures containing multiple brain regions, called cerebral organoids (Lancaster et al., 2013; Lancaster and Knoblich, 2014). This protocol utilizes Matrigel for extracellular matrix support and a rotary bioreactor to significantly extend the culture period, and leverages the intrinsic self-organizing ability of cells to produce organoids that closely mimic brain development and organization. The resulting organoids replicate human-specific features, such as the expanded OSVZ. Over the preceding decade, Lancaster’s protocol has become one of the most widely used methods for culturing 3D brain organoids.

Since then, many researchers have explored various induction protocols. Kadoshima et al. (2013) cultured hESCs in 40% oxygen, supplemented with Y-27632, IWR-1-endo, and SB-431542, to form cortex-like structures with multilayered regions containing the OSVZ. Paşca et al. (2015) developed a new 3D culture method for human cortical spheroids (hCSs) by incorporating dorsomorphin and SB-431542. The hCSs contained mature cortical neurons, resembling the pattern of cortex development, with an expression profile consistent with that of human fetal brain tissue. They also observed mature astrocytes within the hCSs. Beyond innovating culture methods, researchers have sought to improve induction by utilizing novel biological materials or modifying culture bioreactors, among other strategies (Qian et al., 2016; Zhu et al., 2017). These efforts have laid the foundation for advancing brain organoid development and disease modeling. In recent years, as more protocols for regional brain organoids have emerged (Jo et al., 2016; Monzel et al., 2017; Xiang et al., 2021), researchers have begun utilizing these models to mechanistically explore complex neural phenomena and diseases that are discussed in the following sections.

### Classifications and progress of brain organoids

As brain organoid culture methods have progressively advanced, different classifications of brain organoids have emerged. Typically, brain organoids are classified, based on induction protocols, into three categories, namely unguided, guided, and brain organoid assembloids (**Additional Tables 1–3**). Based on the brain regions simulated, they are also classified into specific regional brain organoids (i.e., forebrain, midbrain, hindbrain, and hypothalamus brain organoids).

**Additional Table 1:** Summary of unguided brain organoid protocols

	Cells	ECM	Equipment	Advantage	Limitation	Application	Reference
Cerebral organoids	hESCs; hiPSCs	Matrigel droplets embedded	Spinning bioreactor	Cell type diversity; relying on the cells' self-organizing ability to better simulate development; exhibited typical human behaviors and morphological features	Large batch difference; absence of blood vessels and microglia	Microcephaly	Lancaster et al., 2013; Lancaster and Knoblich, 2014
		No embedding	-	Simplified process; reduce experimental variability; no extrinsic factors; no equipment; larger and better-organized neuroepithelial rosette; no external factors influence tissue self-organization	Absence of blood vessels and microglia; the cultural environment requires a high oxygen level	Zika virus infection	Watanabe et al., 2017
	hESCs	No embedding	-	Relatively simple and inexpensive; no external factors influence tissue self-organization	Absence of blood vessels and microglia; large batch difference; special hESCs culture environment	-	Boisvert et al., 2020
	hiPSCs	Matrigel added into the medium	Orbital shaker	Simplified process	The cultivation scale was limited, with 32 samples per batch	-	Eigenhuis et al., 2023
Forebrain organoids	hESCs	Matrigel droplets embedded	Orbital shaker	Minimal variability, with organoids exhibiting relative uniformity; get forebrain identity with no external factors	Absence of blood vessels and microglia; the process of Matrigel removal demands extensive manual intervention	-	Sivitilli et al., 2020
Telencephalic organoids	hESCs; hiPSCs	Matrigel droplets embedded	Orbital shaker	Reduced batch variance; maintained over extremely long periods; higher maturity; no external factors influence tissue self-organization; much improved organization of neuronal projections	Absence of blood vessels and microglia	-	Giandomenico et al., 2021
Retinal organoid	hiPSCs	Matrigel-coated dishes	-	Microglia are present	Heterogeneity	-	Bartalska et al., 2022
Retinal and cortical organoid	hESCs; hiPSCs	No embedding	Orbital shaker	Mimicking the neuronal projections that connect the eye and brain	Absence of blood vessels and microglia; the efficiency of different cell sources was different	-	Fernando et al., 2022

2D: Two-dimensional; 3D: three-dimensional; EBs: embryoid bodies; ECM: extracellular matrix; hESCs: human embryonic stem cells; hiPSCs: human induced pluripotent stem cells.

**Additional Table 2:** Summary of guided brain organoid protocols

Region-specific	Factor	Cell	ECM	Equipment	Advantage	Limitation	Application	Reference
Brain organoids or cerebral organoids	Dkk-1; LeftyA or SB-431542; BMPRIA-Fc	mESCs; hESCs	No embedding	Culture slide	More effective for inducing the differentiation of cortical cells	Absence of blood vessels and microglia; nutritional deficiency	-	Eiraku et al., 2008
Cortical organoids	IWR1e; SB-431542	hESCs	Matrigel added into the medium	-	Roughly comparable to that in the fetal brain	Limited cell type; the cultivation environment is challenging; high O_2_	-	Kadoshima et al., 2013
	Dorsomorphin; SB-431542	hESCs	Matrigel added into the medium	Orbital shaker	An elongated, continuous, radially organized neuroepithelium; singular continued neuroepithelium structure; rosette structures thicker than cortical organoids; more gradual neural induction	Absence of blood vessels and microglia	-	Pagliaro et al., 2023
	Dorsomorphin; SB-431542	hiPSCs	No embedding	-	More simply; together with deep and superficial cortical neurons	Limited cell type, only excitatory neurons of the dorsal telencephalon	-	Paşca et al., 2015
Forebrain, midbrain and hypothalamus organoids	Forebrain: WNT-3A; A83-01; Dorsomorphin; SB-431542; CHIR99021 Midbrain: LDN-193189; SB-431542; SHH; purmorphamine; CHIR99021; FGF8 hypothalamus organoids: LDN-193189; SB-431542; 1-Thioglycerol; WNT-3A; SHH; purmorphamine	hiPSCs	Diluted Matrigel	Miniaturized spinning bioreactor	Cost effective; the miniaturized equipment	The cultivation environment is challenging	Zika virus infection	Qian et al., 2016
Telencephalic organoids	CHIR99021	hESCs; hiPSCs	Embedded in droplets of Matrigel, added material to the medium, or not embedded	Orbital shaker	-	-	To assess the impact of ECM on human telencephalic organoids	Martins- Costa et al., 2023
Midbrain organoids	Noggin; SB-431542; CHIR99021; SHH-C25II; FGF8	hESCs	Matrigel droplets embedded	Orbital shaker	Possess functional mDA neurons; exhibited biochemical and electrophysiological properties of mature mDA neurons; possess neuromelanin-like granules	-	-	Jo et al., 2016
Ventral midbrain organoids	rhNoggin; SB-431542; CHIR99021; SHH-C24II; FGF-8b	hPSCs	Matrigel droplets embedded	-	Reduced necrosis and supported neuronal maturation; a lower level of variability (spider-silk protein)	Absence of blood vessels and microglia	-	Fiorenzano et al., 2021
Hindbrain organoids	LDN-193189; SB-431542; purmorphamine; RA	hESCs; hiPSCs	Matrigel droplets embedded	Orbital shaker	5-HT-neuron-enriched hindbrain-like organoids	Extended culture durations result in high variability between organoids; limited cell type	-	Valiulahi et al., 2021
Cerebellar organoids	SB-431542; FGF2	hiPSCs	Matrigel droplets embedded	-	Simulate stages akin to the developmental processes of the cerebellum in a physiological embryo	Limited number of identified purkinje neurons; purkinje neurons exhibit limited maturation	-	Nayler et al., 2021
	SB-431542; Noggin; CHIR99021; FGF8b	hESCs; hiPSCs	Matrigel added into the medium	Orbital agitation	Include a human-specific progenitor subtype of the Rhombic Lip; functional Purkinje neurons	-	-	Atamian et al., 2024

5-HT: 5-Hydroxytryptamine; BMPRIA-Fc: bone morphogenetic protein receptor I A fusion protein; DKK-1: Dickkopf-related protein-1; ECM: extracellular matrix; FGF: fibroblast growth factor; hESCs: human embryonic stem cells; hiPSCs: human induced pluripotent stem cells; hPSCs: human pluripotent stem cells; IWRle: IWR-1-endo; mDA: midbrain dopaminergic; mESCs: mouse embryonic stem cells; NT-3: neurotrophin-3; RA: retinoic acid; SHH: sonic hedgehog; SHH-C25II: recombinant mouse sonic hedgehog/shh (C25II) N-terminus; SHH- C24II: recombinant human sonic hedgehog/shh (C24II) N-terminus; WNT-3A: Wnt family member 3A.

**Additional Table 3:** Schematic overview of brain assembloids

Classification	Prat of the assembloids	Simulated interaction	Application	Reference
The interaction between neural cells and nonectodermal cells	Mesodermal progenitor aggregates + neural aggregates	Blood vessels and neural cells	-	Wörsdörfer et al., 2019
	hESCs ectopically expressing hETV2 + cortical organoids	Blood vessels and neural cells	-	Cakir et al., 2019
	HUVECs + cerebral organoids	Blood vessels and neural cells	-	Shi et al., 2020
	Pericyte-like cells + cortical organoid	Perivascular cells within the brain	SARS-CoV-2 infection in CNS	Wang et al., 2021
	PMPs + hPSCs-derived pNPCs	Microglia and neural cells	Zika virus infection	Xu et al., 2021
The interaction between organoids in different regions	Dorsal forebrain organoid + ventral forebrain organoids	Dorsoventral axis of the forebrain; ventral- to-dorsal migration of GABAergic neurons	-	Bagley et al., 2017
	Human subpallium spheroids + human cortical spheroids	Interneuron migration to the cortex	Simulate Timothy syndrome	Birey et al., 2017
	Thalamic organoids + cortical organoids	Thalamocortical projections between human thalamus and cortex	-	Xiang et al., 2019
	Striatal spheroids + cortical spheroids	Corticostriatal projection	Simulate Phelan-McDermid syndrome	Miura et al., 2020
	Hindbrain/cervical spinal cord spheroids + cortical spheroids + skeletal myoblasts spheroids	Cortico-motor circuit	-	Andersen et al., 2020
	Vessel organoids + brain organoids	Neurovascular interactions	-	Sun et al., 2022
	Medullary crucial spinal trigeminal nucleus organoids + thalamic organoids	Trigeminothalamic projection	-	Pang et al., 2024

CNS: Central nervous system; HUVECs: human umbilical vein endothelial cells; hPSCs: human pluripotent stem cells; PMPs: primitive macrophage progenitors; pNPCs: primitive neural progenitor cells; SARS-CoV-2: severe acute respiratory syndrome coronavirus 2.

The generation of unguided/undirected brain organoids relies on the intrinsic ability of stem and progenitor cells to self-organize into complex tissue structures without the addition of guiding small molecules (**Additional Table 1**). With the Lancaster’s protocol, organoids containing distinct yet interdependent brain regions were grown in a simple medium and supported by the extracellular matrix environment of Matrigel (Lancaster et al., 2013). With this protocol, organoids rapidly form various brain regions within 20 to 30 days and can be cultured for up to 10 months in a rotary bioreactor. Lancaster’s method resulted in a more coherent cortical structure, alongside the simultaneous generation of OSVZ regions. The OSVZ, which contains outer radial glia (oRG) and intermediate progenitors, is a characteristic proliferative region of the primate neuroepithelium (Fietz et al., 2010). It plays a critical role in the expansion and gyrification of the human cortex (Sousa et al., 2017). Therefore, the formation of OSVZ regions enhances the utility of brain organoids for studying human neurodevelopment. The key advantage of unguided organoids is their inclusion of diverse cell types, representing forebrain, midbrain, and hindbrain identities within a single organoid, leading to greater complexity. However, unguided organoids rely entirely on self-organization without inductive factors and exhibit significant variability in cell composition across differentiation stages, complicating their use in development and disease modeling (Sidhaye and Knoblich, 2021).

Human brain development adheres to specific biological patterns. Following neurulation, three primary brain vesicles form along the neural tube, giving rise to the entire brain (Accogli et al., 2020). The forebrain subsequently differentiates into the telencephalon, diencephalon, and optic vesicles (Kiecker and Lumsden, 2005; Garcia et al., 2017). This differentiation process is primarily governed by gradient-based signaling pathways. Typically, the generation of specific brain regions in organoids mirrors the developmental processes of actual brain tissue. Following this developmental pattern, researchers generated organoids with specific brain regions by adding morphogens and signaling factors, producing guided brain organoids (**Additional Table 2**). Compared to unguided organoids, guided brain organoids exhibit a more stable developmental trajectory under the control of inductive molecules (Yoon et al., 2019; Eichmüller and Knoblich, 2022). Guided protocols exist for generating brain organoids of specific regions, such as the forebrain (Cederquist et al., 2019), midbrain (Jo et al., 2016; Fiorenzano et al., 2021; Kiral et al., 2023), hindbrain (Valiulahi et al., 2021) and cerebellum (Nayler et al., 2021). Forebrain organoids, particularly cortical organoids, are one of the most extensively studied types of regional brain organoids. A typical induction protocol involves the addition of BMP, TGF-β (dual SMAD inhibition) and WNT pathway inhibitors. For instance, Kadoshima et al. (2013) induced cortical organoids by adding SB431542, a TGF-β/SMAD inhibitor, and IWR-1-endo, a WNT pathway inhibitor. SHH and WNT pathways are the key signaling events directing midbrain formation, while FGF8 is a key molecule that delineates the boundaries between the midbrain and hindbrain (Joksimovic et al., 2009; Sunmonu et al., 2011). Hence human PSCs-derived embryoid bodies can be directed toward a midbrain fate through the activation of SHH and WNT, as well as the addition of FGF8 (Jo et al., 2016). Retinoic acid induces caudalization in a concentration-dependent manner by directly regulating the expression of the homeobox (*HOX*) gene family, and its deficiency results in the failure of hindbrain boundary formation (Cunningham and Duester, 2015; Valiulahi et al., 2021). Muguruma et al. (2015) found that the addition of FGF19 promoted the spontaneous formation of DV polarized neural tube-like structures at the cerebellar level. The combination of FGF19 and stromal cell-derived factor-1 (SDF1) induced continuous neuroepithelial structures in the cerebellar plate. The addition of various morphogens and inductive factors can mitigate batch-to-batch variability to some extent (Velasco et al., 2019). Nonetheless, excessive use of external factors could also disrupt the self-organization and cell–cell interactions, compromising the generation of precise organoids.

As induction protocols for regional brain organoids mature, researchers are attempting to increase organoid system complexity to better recapitulate the intricacies of the human brain. Fusion of brain organoids presents a novel approach (**Additional Table 3**). Assembloids, also referred to as fusion organoids, are created by fusing region-specific organoids (Kanton and Paşca, 2022; Pașca et al., 2022). Researchers place organoids from different regions together such as building blocks, facilitating the connection of neural cells between the organoids. This approach aids in studying the reconstruction of neuronal circuits and the transmission of information between neurons (Xiang et al., 2019; Chen et al., 2020). During fetal neurodevelopment, interneurons originating from the medial ganglionic eminence migrate tangentially to specific functional cortical sites (Lim et al., 2018). This process is essential for cortical maturation. Birey et al. (2017) generated human subpallium spheroids by adding inhibitor of WNT production-2 (IWP2) and smoothened agonist (SAG), and fused them with hCSs to replicate neuronal migration to the cortex. During brain development, neurons establish thalamocortical axonal projections (Harris et al., 2019). Miura et al. (2020) fused striatal spheroids with cortical spheroids to replicate striatum-cortical projections *in vivo*. To reconstruct the DV axis, Bagley et al. (2017) induced dorsal and ventral forebrain organoids, encapsulated them in Matrigel, and fused them in culture, thereby recapitulating robust, directed migration of GABAergic interneurons from ventral to dorsal forebrain organoids. Beyond reconstructing neural projections and circuits within distinct brain regions, researchers have also attempted to replicate connections between the brain and other organs (Andersen et al., 2020; Pang et al., 2024). Andersen et al. (2020) sought to replicate cortical motor loops by co-culturing hCSs and spinal spheroids, subsequently linking them with skeletal muscle spheroids to generate 3D cortico-motor assembloids. They found that cortical neurons in assembloids projected to and connected with skeletal muscle spheroids, while spinal-derived motor neurons formed connections with muscle tissue. Assembloids can also be utilized for disease modeling, such as brain organoids derived from hESCs or patient iPSCs co-cultured with patient-derived glioma stem cells to form 3D organoids for glioma studies (Krieger et al., 2020; Pawlowski et al., 2023).

The interaction between neural cells and non-ectodermal cells is increasingly recognized as a crucial factor in neurodevelopment and diseases. To model this compositional diversity of the developing human brain, non-ectodermal cell types or their progenitors were introduced into brain organoids at various stages of differentiation to create multi-lineage assembloids. For example, the addition of mesodermal cells (Wörsdörfer et al., 2019; Wang et al., 2021), endothelial cells (Shi et al., 2020), or stem cells expressing lineage-switching transcription factors can lead to the formation of vascularized brain organoids (Cakir et al., 2019). Researchers have also attempted to generate brain organoids containing microglia, specialized immune cells within the brain, through directly incorporating primitive macrophage progenitors (Xu et al., 2021). Numerous studies have highlighted the close relationship between vascular tissue and microglia, indicating that the appearance of microglia accompanies the formation of vascularized brain organoids (Sun et al., 2022). Besides microglia, mature oligodendrocytes are rarely present in brain organoids, as gliogenesis occurs after neurogenesis (Alvarez-Buylla et al., 2001). Researchers have therefore attempted to promote oligodendrocyte formation within brain organoids. Marton et al. (2019) developed methods to differentiate hiPSCs into human oligodendrocyte brain organoids by adding oligodendrocyte proliferation- and maturation-promoting factors (e.g., platelet-derived growth factor [PDGF], hepatocyte growth factor [HGF], insulin-like growth factor 1 [IGF1]) to facilitate human oligodendrocyte spheroids from brain organoids. Biochemical analysis and single-cell RNA sequencing confirmed the presence of mature oligodendrocytes.

The development of complex organoids has significantly reduced the challenges to model complex physiological phenomena and diseases. *In vitro*, brain organoids with distinct regional characteristics can be more easily manipulated, overcoming many technical obstacles *in vivo*. To better model complex neural development and diseases, constructing complex brain organoids represents a key future direction, while cell-organoid co-culture models could simplify and facilitate deeper insights into cellular interactions and disease mechanisms. Precise control of the number and proportion of cells and cell types is crucial, emphasizing the need for relevant research. Despite their complexity, brain organoids are simplified 3D tissues and cannot fully replicate the precise temporospatial structure *in vivo*, posing intrinsic caveats for accurate physiological and pathological modeling.

To address the limitations of brain organoids’ simplicity, numerous studies have explored transplanting brain organoids into living organisms. Mansour et al. (2018) transplanted cerebral organoids derived from hESCs into the retrosplenial cortex of immunodeficient mice, successfully developing an efficient *in vivo* engraftment model. The transplanted organoids integrated well into the mouse brain, exhibiting more mature neural differentiation patterns and forming extensive vasculature. Schafer et al. (2023) co-cultured yolk-sac erythromyeloid progenitors with cortical brain organoids to create a neuroimmune organoid model, which they subsequently transplanted into the retrosplenial cortex of immunocompromised NOD/SCID mice. The researchers observed that the transplanted cells exhibited more mature morphology, long-term survival of human microglia, and progression through the developmental process to acquire immune function. They also utilized this model to investigate the effects of the brain environment and microglia on autism spectrum disorder (ASD). Intracerebral transplantation methods have successfully formed complex brain organoids containing blood vessels and microglia (Wang et al., 2023).

## Applications of Brain Organoid Technology

### Study of human-specific brain development

Human neural development is a highly regulated and dynamic process, initiating with the emergence of neuroectodermal cells and extending well beyond birth. During weeks 3–4 of gestation, the neuroectoderm cells thicken and fold, leading to the formation of the neural tube. Following the completion of neurulation, NPCs within the neural tube are patterned along the AP and DV axes, influenced by morphogen gradients (Cadwell et al., 2019). Furthermore, the process of neurodevelopment extends into the first few years after birth, during which synapse formation and myelination continue, ultimately leading to the establishment of complete and complex neural circuits (Stiles and Jernigan, 2010). Neural development is closely tied to spatial and extracellular matrix signals, which are often lost in 2D cell culture models. Several studies have highlighted the differences between human and rodent neural development. Structurally, the folding of the human cerebral cortex markedly increases the cortical surface area, largely due to the presence of OSVZ (Del-Valle-Anton and Borrell, 2022). Single-cell sequencing data revealed that many brain cell types are conserved between humans and mice (Bakken et al., 2021). However, significant differences exist in cell proportions, gene expression levels, and hierarchical distributions (Hodge et al., 2019). As a result, rodent models fail to accurately mimic many aspects of human physiology. Although nonhuman primate models can replicate human cognitive, behavioral, and social traits, they are prohibitively expensive for most laboratories. Additionally, the use of nonhuman primates in research poses significant ethical concerns.

**Figure 2:**
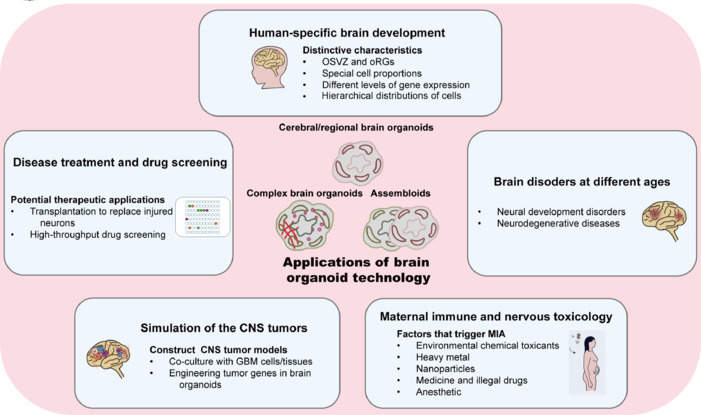
Summary of the application of brain organoids. Brain organoids have diverse applications, including the exploration of human brain development and neurodevelopmental disorders. They are also valuable for investigating maternal immune activation toxicology and CNS tumors. Owing to their ability to be mass-produced *in vitro*, brain organoids are particularly advantageous for disease treatment research and drug screening. CNS: Central nervous system; GBM: glioblastoma; MIA: maternal immune activation; oRG: outer radial glial cell; OSVZ: outer subventricular zone.

Brain organoids have emerged as a powerful model for studying neural development at the fetal stage rather than the postnatal or adult stages (**Figure 2**; Velasco et al., 2019; Li et al., 2024). For example, the cerebral cortex is located on the dorsal side of the telencephalon and is the outermost structure of the brain’s neural organization. The six-layered structure of the human neocortex is conserved during development, with neural cells migrating in an inside-first, outside-last pattern to form the cortex (Vanderhaeghen and Polleux, 2023). The innermost layer is the multiform layer (lamina VI), while the outermost is the molecular layer (lamina I). This I–VI layers structure is replicated in both guided and unguided brain organoids (Lancaster et al., 2013; Otani et al., 2016). Many protocols produced self-organizing cortical structures containing human-specific oRG progenitors, primarily located in the OSVZ, offering compelling evidence of organoids’ capacity to mimic human neurodevelopment. Single-cell sequencing, epigenetic analyses, and spatial transcriptomics have also demonstrated that cell diversification in organoids is intricately linked to the endogenous processes of human brain development (Uzquiano et al., 2022) and enabled the study of developmental trajectories of various neural subtypes, allowing comparisons with human brain data (Fiorenzano et al., 2021). Nonetheless, compared to actual brain tissue, the cortical structure of cerebral organoids remains imperfect, with notable differences in layer thickness and cell distribution. In addition, brain organoids rarely contain endothelial cells, microglia, or oligodendrocytes (Di Stefano et al., 2025). These limitations prevent organoids from reproducing certain neurodevelopmental phenomena, such as the cortical folding, and neurovascular unit and function (Di Lullo and Kriegstein, 2017).

Brain organoids are mainly suitable to simulate early embryonic brain development, largely due to poor nutrition and oxygen supply; this significantly limits their application scope (Tanaka et al., 2020). Studies have employed bioreactors or organoid slicing to modify conditions, enhancing neuronal maturation and long-term survival *in vitro* (Qian et al., 2018, 2020; Suong et al., 2021; Szebényi et al., 2021). Bioreactors suspend organoids in a liquid environment and allow the medium to flow or be agitated, thereby increasing nutrient and oxygen intake and reducing the risk of necrosis at the center of the organoid. Consequently, bioreactors enhance the homogeneity of brain organoids and reduce variability introduced by batch-to-batch and operational differences (Qian et al., 2016), which is crucial for large-scale cultures and applications. Nonetheless, the influence of mechanical force should not be ignored. Bioreactors have a hydrodynamic effect on organoids, and different mixing methods would affect the development of brain organoids (Suong et al., 2021). Organoid slicing is another straightforward method to improve oxygen supply, similar to the classic organotypic slice culture technique at the air–liquid interface. Similar to processing rodent brain samples, researchers embedded organoids in 3% low-gelling temperature agarose, sliced them into 300–500 μm sections using a vibrating microtome, and cultured them in plates (Giandomenico et al., 2019). This culture method promotes abundant axonal growth, resembling nerve bundles *in vivo* (Qian et al., 2020; Giandomenico et al., 2021).

The orderly arrangement of neurons facilitates the formation of intricate connections, weaving a complex neural network. Electrical signals propagate through neural networks to regulate a wide range of life activities. Biochemical analysis of brain organoids only partially validates their use as models of neural development without adequately addressing the presence of neural networks and electrical activity (Samarasinghe et al., 2021; Tasnim and Liu, 2022). Patch-clamp recording, calcium imaging, and multi-electrode arrays are commonly used electrophysiological techniques. Calcium imaging can monitor the electrical activity of hundreds or thousands of neurons, and neurons in 3D brain organoids, differentiated for 40–50 days, exhibit calcium signaling (Xiang et al., 2017). Multi-electrode array is a technique for extracellular recording of electrical signals from neurons or other excitable cells, using arrays of electrodes to enable long-term monitoring of electrical properties in neural networks derived from tissues or stem cells (Spira and Hai, 2013). Researchers can also place brain organoids on multi-electrode arrays to measure electrical activity and track the emergence and maturation of various electrophysiological properties over long-term observations (Fair et al., 2020; Huang et al., 2022a; Ma et al., 2024).

### Study of brain disorders

Numerous neurological diseases, including neurodevelopmental disorders (NDDs), neurodegenerative diseases, and brain tumors, are linked to genetic mutations (Klingler et al., 2021; Manoli and State, 2021). The prevalence of these diseases is notably high in the general population. In 2018, 2.3% of 8-year-old children in the United States of America were diagnosed with ASD (a NDD) (Hirota and King, 2023). In 2021, an estimated 55.2 million people worldwide were living with dementia, a condition broadly associated with many neurodegenerative and neurological diseases such as Alzheimer’s disease and the numbers were projected to increase to 78 million by 2030 and to 130 million by 2050 (Long et al., 2023; Jiang et al., 2025). At present, the pathogenesis of these diseases remains unclear and there is no cure for most of them, with treatments focused solely on symptom alleviation and delaying disease progression. Hence, there is an urgent need to investigate the pathogenesis and develop new treatment options. In many cases, therapies developed from animal models show limited efficacy in humans, highlighting the need for more reliable models. Brain organoids, due to their close resemblance to human brain tissue, offer promise.

#### Simulation of neural development disorders

NDDs (e.g., ASD, intellectual disability, attention deficit hyperactivity disorder, and epilepsy) (Thapar et al., 2017) are often caused by genetic errors, affecting the central nervous system development through impairing motor skills, cognition, communication, and behavior (Parenti et al., 2020). Brain organoids, as *in vitro* models, are highly suitable for genetic manipulation, and their mass culture facilitates pathogenic gene screening. Comparison of gene expression profiles of human brain tissues and brain organoids confirmed that organoids closely mimic the genetic features of early embryonic development (Camp et al., 2015), suggesting that brain organoids are highly suitable for NDD modeling. Numerous studies have demonstrated that NDD brain organoids developed patient-like phenotypes, validating their utility to mimic disease characteristics. Zhang et al. (2020) developed a *Rab39b*-mutant organoid model in which NPC over-proliferation led to an increase in organoid size, replicating the macrocephaly observed in *Rab39b*-mutant patients. Mariani et al. (2015) employed telencephalic organoids to study neurodevelopmental changes in patients with ASD. The brain organoids demonstrated an accelerated cell cycle and overproduction of GABAergic inhibitory neurons, with forkhead box G1 (FOXG1) overexpression driving increased inhibitory neuron production. Jourdon et al. (2023) selected 13 ASD macrocephalic or normocephalic patients and used iPSCs from patients and pairing fathers to generate forebrain organoids. Single-cell transcriptomics of these organoids revealed an imbalance in excitatory cortical neuron subtypes during early neurogenesis in patients with ASD and suggested that distinct pathogenic mechanisms exist between patients with and without macrocephaly.

Beyond morphological differences, patients with NDDs often exhibit disordered neuronal firing, leading to symptoms (e.g., epilepsy), and patient-derived brain organoids can also replicate this abnormal electrical activity. For example, Birey et al. used cells from patients with Timothy syndrome to generate pallium-subpallium assembloids for calcium imaging experiments (Birey et al., 2017). Compared to the control group, assembloids derived from patients with Timothy syndrome increased jump frequency, significantly reduced jump length, and decreased movement velocity, replicating the abnormal neuronal firing observed in patients with Timothy syndrome. The factors contributing to NDDs are complex and multifaceted. Evidence suggests that exposure to risk factors during pregnancy can contribute to NDD development, and brain organoids can be used to assess these exposure risks (Jiang et al., 2016). The details of maternal immunity are also discussed in this review.

#### Modeling neurodegenerative diseases

Neurodegenerative diseases are chronic conditions characterized by progressive loss of neuronal function and structure (Wilson et al., 2023). Several studies indicated that mice may not be suitable models for studying neurodegenerative diseases. For instance, there is limited homology between proteins in mice and humans, and mice lack orthologs of several risk genes linked to neurodegenerative diseases (Breschi et al., 2017). Consequently, researchers have begun utilizing brain organoids to model neurodegenerative diseases. Alzheimer’s disease (AD) is the most common neurodegenerative disorder, including familial and sporadic forms (Sabate-Soler et al., 2024). Both forms of AD are often characterized by amyloid accumulation, hyperphosphorylated tau protein, and neurodegeneration or neuronal injury (Jack et al., 2018). Raja et al. (2016) successfully reproduced these pathological features and validated treatment regimens using iPSCs derived from patients with AD. As demonstrated by enzyme-linked immunosorbent assay, the brain organoids derived from AD patient iPSCs showed significantly higher levels of amyloid-beta peptides in culture medium compared to controls, and these levels progressively increased with time in culture. This model also presented hyperphosphorylated tau and endosome abnormalities. The beneficial effects of β- and γ-secretase inhibitor treatment were also demonstrated with the organoids. It was initially thought that modeling late-onset familial AD with organoid cultures would be challenging. Nevertheless, emerging studies suggested that such modeling is possible. Long-term culture of brain organoids led to the development of extracellular amyloid plaques and intracellular neurofibrillary tangles associated with late-onset AD (Gonzalez et al., 2018). Chen et al. (2021b) exposed sporadic AD patient-derived iPSCs brain organoids to serum to mimic blood-brain barrier disruption, a potential pathogenic factor in AD, and recapitulated AD-like pathology. Parkinson’s disease is another neurodegenerative disorder. Similarly, many investigators have used midbrain organoids to model Parkinson’s disease, which offer the capability to reconstruct dopamine neuronal networks and recapitulate their deficits (Fiorenzano et al., 2021; Mohamed et al., 2021).

#### Study of maternal immunity and neural toxicities

The maternal–fetal interface acts as a critical defense barrier, protecting the fetus from pathogens while facilitating the transfer of maternal antibodies (Erlebacher, 2013). Viral infections or exposure to environmental toxins during pregnancy can trigger maternal immune activation in pregnant women. Epidemiological and neurobiological studies indicated that maternal immune activation and the resulting inflammation can disrupt fetal nervous system development, potentially leading to NDD-like autism (Han et al., 2021). As research progresses, it has become increasingly evident that the preconception environment plays a critical role in fetal development. Rodent models remain the primary tools for studying maternal immune activation, typically induced using viral mimetics, such as polyriboinosinic-polyribocytidylic acid, synthetic double-stranded RNA, or lipopolysaccharide (Murray et al., 2019). However, the extent to which these rodent models mimic human maternal immune activation remains uncertain. Brain organoids offer a valuable alternative for studying infectious diseases, such as microcephaly resulting from Zika virus infection (Qian et al., 2016; Li et al., 2017; Xu et al., 2021). Viral infection of brain organoids is easier to manage, enabling detailed observation of the effects of Zika virus on neurodevelopment over time (Li et al., 2017). Interleukin-6 (IL-6) in the prenatal brain, primarily maternally derived, participates in the IL-6 signal transducer/Janus kinase/signal transducer and activator of transcription (IL6ST/JAK/STAT) signaling pathway and contributes to neocortical development (Sarieva et al., 2023). Introduction of IL-6 and soluble IL-6 receptor chimeric proteins to 45-day dorsal forebrain organoids, a stage corresponding to the first and second trimester neocortex development, affected the number of radial glial cells and disrupted cortical neuron migration and organization. Treatment of brain organoids with tumor necrosis factor alpha disrupted the formation of brain rosettes and was associated with a scar-like tissue similar to the gliosis-like response observed *in vivo* (Benson et al., 2020).

Fetal brain development is particularly susceptible to common environmental toxins, such as bisphenol A (Bansal and Zoeller, 2019) and heavy metals (Wylie and Short, 2023). Exposure to environmental toxins during pregnancy or postpartum has been linked to neurological disorders and neurodegenerative diseases (Nabi and Tabassum, 2022). We have recently utilized brain organoid models and showed that bisphenol A exposure caused severe cortical developmental abnormalities, including inhibited progenitor cell proliferation, premature neuronal differentiation, and abnormal cortical layering and structural organization (Cao et al., 2023). We have also employed a brain organoid model to simulate the neurotoxic effects of environmental cadmium on brain development (Hu et al., 2025). Our work confirmed that brain organoids can serve as an easy and powerful model for simulating the neurotoxicity of environmental toxins. Rotenone, a key component of common broad-spectrum insecticides, exhibits both neurotoxic and reproductive toxic effects. A recent study indicated that rotenone induces ferroptosis in mice, leading to abnormal methylation in mouse brain organoids and the loss of normal neuronal function (Huang et al., 2022b). Albeit important, studies related to environmental toxicological effects on pregnant women and fetuses remain limited. The mechanisms underlying the neural toxicity of numerous environmental toxins and contaminants warrant further investigation. In this area, modeling with human brain organoids may be both crucial and fruitful. Existing brain organoid models offer the advantage of a 3D environment. Nonetheless, it is important to develop brain organoids with more refined regional characteristics for better toxicological studies since many damages are associated with specific cell types and brain regions.

#### Simulation of central nervous system tumors

Central nervous system tumors, classified into gliomas, glioneuronal tumors, and neuronal tumors, are common across all age groups (Berger et al., 2022; Cai et al., 2025). Adult-type diffuse gliomas are the most common and aggressive forms of gliomas; among them, glioblastoma (GBM) is linked to the highest mortality rate. Heterogeneity is increasingly considered the most important factor underlying GBM complexity and drug resistance (Brennan et al., 2013; Neftel et al., 2019). Addressing this heterogeneity with an appropriate model is crucial for both mechanistic exploration and drug development. Hubert et al. (2016) developed the first GBM organoid model by dissociating tumor tissues into single cells and encapsulating them inside a cellular matrix. Jacob et al. (2020) developed an alternative method involving GBM organoids derived directly from patient tumor tissues without enzyme digestion. These organoids were obtained by fragmenting the tumor into small tissue blocks and culturing them in suspension to form circular organoids. While patient-derived models preserve the inter- and intra-tumor heterogeneity, co-culturing brain organoids with GBM organoids provides a brain-like environment and an invasive scaffold, more closely mimicking physiological conditions. In current co-culture models, GBM cells adhere to and gradually invade brain organoids. They more accurately mimic the phenotypes observed in the donor patients and enable better and easier microscopic observation (Linkous et al., 2019; Krieger et al., 2020). Zhang et al. (2021) co-cultured excised glioma tissues with brain organoids and in some of the co-cultures identified efficient tumor integration and infiltration into brain organoids and typical patient tumor features. An alternative approach involves engineering brain organoids to delete tumor suppressor genes or overexpress genes associated with brain tumors (Bian et al., 2018; Azzarelli, 2020). This strategy allows for the observation of de novo tumorigenesis in the same culture and a better study of interactions between transformed and non-transformed cells. The glioma and brain organoids co-culture model could have significant advantages over other glioma models (Pine et al., 2020). In their study, Pine et al. (2020) compared four different models, namely 2D glioma sphere cultures, tumor organoids, GBM cerebral organoids, and *in vivo* transplantation models. RNA sequencing analysis revealed that glioma stem cells in GBM cerebral organoid models showed a higher transcriptomic correlation with corresponding patient samples compared to other models.

### Brain organoids for disease treatment and drug screening

The use of brain organoids in disease research extends beyond modeling and includes potential therapeutic applications. Since brain tissue has limited regenerative capacity, one key research focus is the discovery of methods to replace damaged neurons. Transplantation of 2D-differentiated neural cells into injury sites to replace damaged neurons, aiming to reconnect them with existing neurons and restore neural networks, has been attempted in numerous studies (Xiong et al., 2021). The use of brain organoids for *in vivo* transplantations and disease therapy could also be explored (Dong et al., 2021). Compared to 2D-cultured neural cells, organoid-derived neurons are more mature with greater cellular complexity and capacity to form neural unit structures (Revah et al., 2022; Jgamadze et al., 2023; Xu et al., 2023). Hypothetically, organoid transplantation could be effective in treating neurological diseases.

Drug development is a lengthy and costly process, involving multiple stages of identification, preclinical testing, and clinical trials. Brain organoids offer advantages in high-throughput screening, enabling the use of multiple patient samples and producing results that more accurately reflect human physiological conditions (Park et al., 2021). Apart from target organs, drugs often affect other organs; therefore, a comprehensive understanding of systemic effects is necessary. Creating accurate simulations of human organ systems is thus important for drug screening studies. Integrated systems, known as organ-on-a-chip platforms, combine multiple 3D organoids, including brain organoids. These systems can be used to simulate the physiological responses of multiple organs to drugs and assess potential drug toxicity (Skardal et al., 2020; Ingber, 2022).

Although brain organoids have been increasingly employed in studies of neurodevelopment, neurological diseases, and related drug development, their current cellular limitations remain a significant challenge that may impact research outcomes. For instance, brain organoids often lack microglia and vascular structures, resulting in an altered immune environment and aberrant gene expression (Andrews and Kriegstein, 2022). The application of genome-editing technologies can facilitate the introduction of non-ectodermal lineage cells, thereby enhancing the cellular complexity of brain organoids, as elaborated in the section ‘Increasing the complexity of organoids’ below.

## Genome Editing in Neurodevelopment and Disease Studies

**Figure 3:**
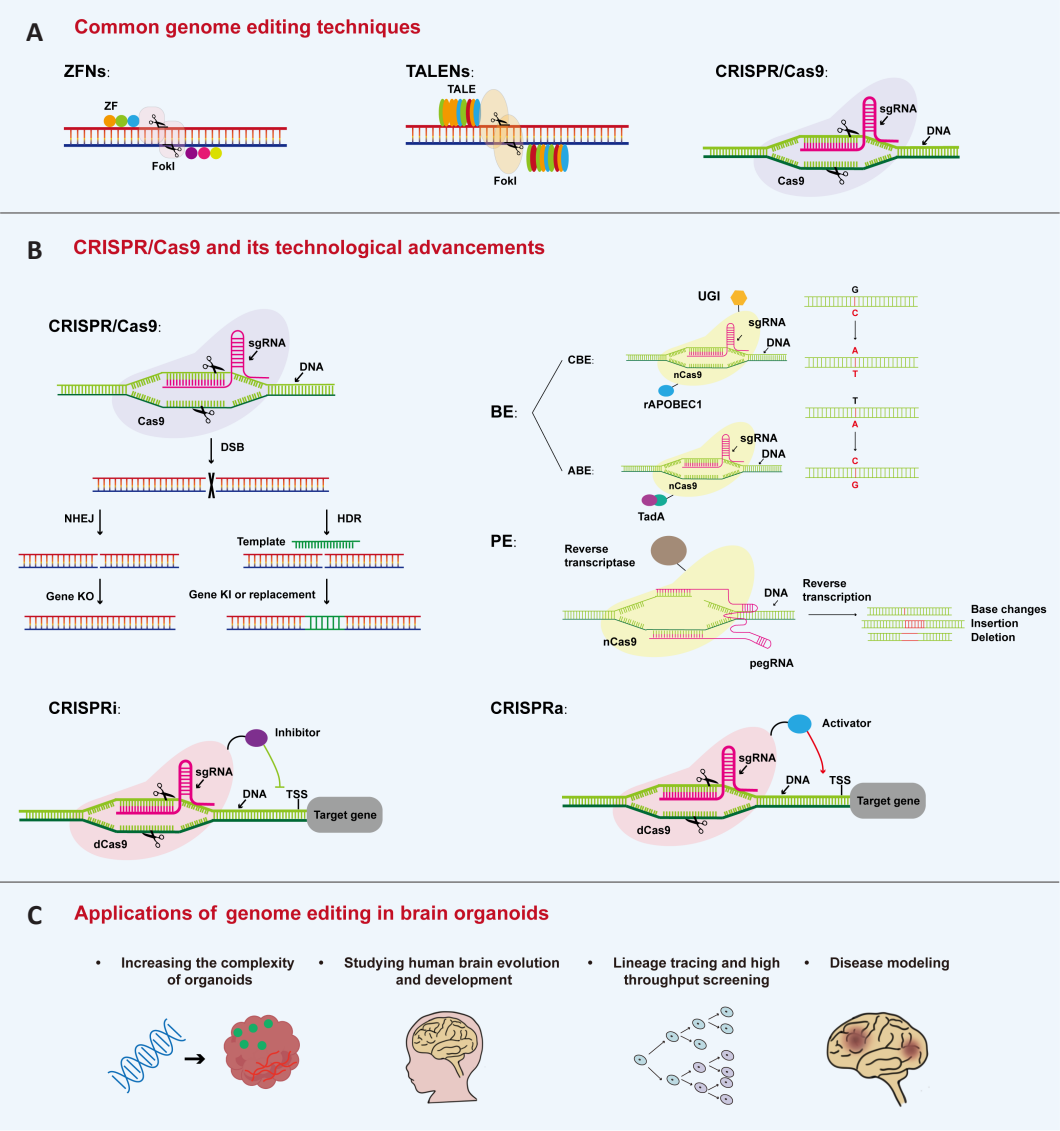
Applications of CRISPR/Cas9 in brain organoids. (A) The currently widely used genome editing technologies include ZFNs, TALENs, and CRISPR/Cas9. CRISPR/Cas9 technology has become the dominant gene editing tool because of its ease of use, low cost, and rapid execution. The mechanism of CRISPR/Cas9 involves the use of sgRNA to direct the Cas9 protein to a specific target site, where it introduces DSBs. In response, the cell activates its DNA repair pathways, primarily via NHEJ or HDR. (B) Recently, BE and PE have been developed as advancements in the CRISPR/Cas9 system. In BE, the Cas9 variant nCas9 induces a single-strand break, followed by the action of deaminases—commonly adenine or cytosine deaminase—to convert specific bases, resulting in precise single-base edits. In contrast, PE modifies the sgRNA to form pegRNA, which, after nCas9 induces a single-strand break, is used by reverse transcriptase to incorporate the desired sequence directly into the DNA strand. The CRISPRi and CRISPRa systems represent emerging technologies based on CRISPR/Cas9, enabling the regulation of target gene activation and inhibition through the binding of deactivated Cas9 to the transcription start site of the target gene. (C) The integration of genome editing technologies with brain organoids holds significant potential for advancing research. Complex brain organoid models can be developed to investigate human neurodevelopment and associated diseases while enabling lineage tracing and high-throughput screening. ABE: Adenine base editor; BE: base editor; Cas9: CRISPR-associated nuclease 9; CBE: cytosine base editor; CRISPR/Cas9: clustered regularly interspaced short palindromic repeat/CRISPR-associated nuclease 9; CRISPRa: CRISPR activation; CRISPRi: CRISPR interference; DSBs: double-strand breaks; dCas9: dead Cas9; HDR: homology-directed repair; KO: knockout; KI: knock-in; NHEJ: nonhomologous end joining; nCas9: nickase Cas9; PE: prime editing; pegRNA: prime editing guide RNA; rAPOBEC1: rat apolipoprotein B mRNA editing enzyme catalytic polypeptide 1; sgRNA: single guide RNA; TadA: escherichia coli tRNA adenosine deaminase; TALE: transcription activator-like effector; TALENs: transcription activator-like effector nucleases; TSS: transcription start site; UGI: uracil DNA glycosylase inhibitor; ZF: zinc finger; ZFNs: zinc-finger nucleases.

Genome editing technologies represent a breakthrough that has been considerably accelerating the study of the human neural system and diseases. Presently, the most commonly used genome editing system is the type II CRISPR/Cas9 system derived from *Streptococcus pyogenes* (Wang et al., 2022). Prior to the advent of CRISPR/Cas9 technology, the dominant genome editing tools were primarily zinc-finger nucleases and transcription activator-like effector nucleases. However, CRISPR/Cas9 has progressively become the preferred method among researchers in the field of stem cell biology due to its simplicity and higher efficiency (Matsumoto and Nomura, 2023; Pacesa et al., 2024). Numerous articles provide detailed summaries and explanations for genome editing technologies, including the history, principles, and technical proceedings and advances (Hanna and Doench, 2020; Kampmann, 2020; Pacesa et al., 2024; Villiger et al., 2024). Below, we provide a brief overview of various genome editing techniques, followed by a detailed summary of the applications of CRISPR/Cas9 in brain organoids (**Figure 3** and **Additional Table 4**; Lai et al., 2024).

**Additional Table 4:** Summary of commonly used genome editing and related gene expression manipulation techniques

Technology	Principle	Endonuclease	Advantage	Limitation	Application in brain organoid
ZFNs	DSBs	FokI	A minimal number of off-target breaks; small protein size	High cost; time consuming; increased synthesis complexity	-
TALENs	DSBs	FokI	Enhanced specificity; efficient in producing DSBs; a minimal number of off-target breaks; precisely recognize the site	High cost; time consuming; larger DNA sequence recognition size; large protein size	-
CRISPR/Cas9	DSBs	Cas9	Relatively cheap and easy to operate	High off-target rate; preferentially applicable to proliferating cells; large protein size and Cas9 toxicity	Trujillo et al., 2021; Cakir et al., 2022
BE	DNA single-strand break; mismatch repair	-	Greater efficiency; simple process; without requiring DSBs or donor DNA; high accuracy; low cytotoxicity; fewer byproducts	The range of applications is limited	-
PE	DNA single-strand break; mismatch repair	-	Without requiring DSBs or donor DNA; it can perform the deletion and insertion of various bases; much lower off-target; fewer byproducts	More challenging to design and deliver compared to BE	-
CRISPRi	dCas9 protein binds to the TSS of the target gene, thereby inhibiting transcription and suppressing gene expression without altering the genomic sequence	-	Process is reversible and does not alter the genome; multiple sgRNAs can be used for screening; transcription level prevents the initiation of transcription	May lead to nonspecific inhibition; different genes exert varying effects; bidirectional promoters should be considered; genes with multiple start sites require multiple sgRNAs	Cakir et al., 2022; Johansson et al., 2022; Lai et al., 2024
CRISPRa	dCas9 protein to activate gene expression by binding to the TSS of a target gene without altering the genome	-	Process is reversible and does not alter the genome; can simultaneously target multiple genes; not affected by long ORFs; no affected by splice isoforms	May lead to nonspecific activation; bidirectional promoters should be considered	Chen et al., 2024

BE: Base editors; CRISPR/Cas9: clustered regularly interspaced short palindromic repeat/CRISPR-associated nuclease 9; dCas9: dead Cas9; DSBs: double-strand breaks; ORFs: open reading frames; PE: prime editors; sgRNA: single-guide RNA; TALENs: transcription activator-like effector nucleases; TSS: transcription start site; ZFNs: zinc-finger nucleases.

Through continuous refinement, researchers have been simplifying and improving the CRISPR/Cas9 system since its development, leading to its widespread adoption in numerous laboratories. The core principle involves using engineered nucleases to create DNA double-strand breaks (DSBs) at specific genomic sites with cellular repair mechanisms activated. In the absence of a template, the cell employs non-homologous end joining to either delete or insert a sequence at the target site. Alternatively, homologous directed repair allows for precise gene integration or base correction when a donor template homologous to the target chromosomal locus is available (Ma et al., 2014; Adli, 2018). Owing to the advantages of CRISPR/Cas9, the field has rapidly expanded beyond conventional genome editing with the advent of base editing (BE) and prime editing (PE) technologies (Testa and Musunuru, 2023). Both approaches offer distinct advantages and are appropriate for a variety of applications. BE enables the direct replacement of a target base with the desired nucleotide without requiring DSBs or a donor template (Komor et al., 2016; Nishida et al., 2016; Gaudelli et al., 2017). There are two main types of base editors, namely the cytosine base editor, which replaces cytosine with thymine, and the adenine base editor, which replaces adenine with guanine. The glycosylase base editor has enabled the conversion between cytosine and guanine, thereby expanding the capabilities of the BE system (Hendriks et al., 2020; Kurt et al., 2021). CRISPR/Cas9-mediated BE process is relatively straightforward and exhibits high efficiency; this approach is particularly suitable for investigating genetic diseases resulting from point mutations (Rees and Liu, 2018; Tanaka and Chung, 2025). For instance, Geurts et al. (2023) introduced cancer-related genetic mutations into hepatocyte, endometrial, and intestinal organoids, representing an important advancement in the application of organoid models for cancer research. PE technique utilizes a catalytically impaired Cas9 endonuclease fused to an engineered reverse transcriptase, with the PE guide RNA both specifying the target site and encoding the desired genetic alteration. PE eliminates the requirement for DSBs and donor DNA, thereby simplifying the preparation of the editing components and enhancing editing efficiency and precision (Anzalone et al., 2019; Chen and Liu, 2023). PE has increasingly shown high potential for the study and treatment of genetic disorders (Zhao et al., 2023). Examples include the correction of the A•T-to-T•A transversion mutation in the hemoglobin subunit beta (*HBB*) gene, which causes sickle cell disease, and the insertion of 4 bp into the hexosaminidase subunit alpha (*HEXA*) gene, which leads to Tay–Sachs disease (Anzalone et al., 2019). Clearly, there is considerable promise for research aimed at harnessing this advanced gene editing technology in organoid models. Nonetheless, studies exploring the application of BE and PE in organoids remain scarce (Schene et al., 2020; Bulcaen et al., 2024). Other noteworthy technologies are the CRISPR interference (CRISPRi) and CRISPR activation (CRISPRa) systems, both mainly based on CRISPR/Cas9 (Gilbert et al., 2013; Qi et al., 2013). These gene expression regulatory systems utilize nuclease-dead mutants of the Cas9 protein (dCas9). Due to the lack of DNA cleavage activity, these mutants do not alter the genome sequence; however, they are capable of binding to the transcription start site of the target gene (Kampmann, 2018). In the CRISPRi system, dCas9 inhibits gene expression by preventing the binding of transcription factors and RNA polymerase. In contrast, in the CRISPRa system, dCas9 is fused to transcriptional activators, which bind to the transcription start site to enhance gene expression. CRISPRi and CRISPRa technologies can also be integrated with a high-throughput sequencing approach termed CRISPR screening, which enables the identification of a broad spectrum of potential gene targets or candidate disease-associated genes.

Gene editing enables precise genome modifications and studies of gene functions, interactions, and regulations. With the help of gene editing, researchers can create disease models enabling mechanistic studies and precisely identify the underlying genes and/or molecular pathways (Zheng et al., 2023). When combined with high-throughput screening and sequencing, genome editing could greatly facilitate the identification of pathogenic genes in complex, multi-gene neurological diseases, while enabling drug testing in diverse genetic environments and/or patient-specific backgrounds (Oldrini et al., 2018). Modern genome editing technology permits lineage tracing, which can be relatively easily utilized to elucidate the origin and fate of neural cells in an unprecedented depth, resolution, and scale, as well as identify numerous novel research opportunities (He et al., 2022; Fleck et al., 2023; Li et al., 2023). The combination of genome editing with human brain organoids is gaining momentum lately, unleashing the unprecedent technical potential to enable and accelerate the study of human brain development and diseases (**Figure 3**).

### Increasing the complexity of organoids

The limited diversity of cell types represents a major technical bottleneck in brain organoid construction and modeling. Cells in brain organoids generated by existing protocols are mainly from ectodermal lineages, significantly limiting their utility to mimic physiological states (Andrews and Kriegstein, 2022). In particular, microglia, the primary immune cells of the brain but completely missing in most organoid models, are indispensable for human brain development, function, and homeostasis. During embryogenesis, microglia originate from yolk sac progenitors and migrate to the brain at embryonic day 8.5 in mice or gestational weeks 4.5–5.5 in humans (Paolicelli et al., 2022). As a result, microglia are rarely found in either region-specific or unguided brain organoids. However, they are important for brain organoid development, facilitating their maturation (Park et al., 2023). Through overexpressing the hematopoietic transcription factor PU.1 in ESCs and mixing 10% PU.1-overexpressing cells with wild-type stem cells, Cakir et al. (2022) successfully created cortical brain organoids containing microglia, known as microglia-containing human cortical organoids. This strategy can produce a significant amount of microglia, while enabling easy adjustment of their numbers in the organoids. In combination with CRISPRi, microglia-containing human cortical organoids produced by this strategy were successfully used for AD-related microglia gene research. Nevertheless, the absence of vasculature in these organoids results in central necrosis, causing premature microglia activation and organoid maturation. In addition, the distribution of microglia in microglia-containing human cortical organoids is uncontrollable and may not resemble that observed *in vivo*.

Vascularization is another key issue for brain organoid construction and modeling. Blood vessels are the first organs formed during embryonic development, responsible for transporting oxygen and nutrients and are crucial for the average growth and function of the brain (Chung and Ferrara, 2011). Using a similar strategy of ectopically expressing human E26 transformation-specific variant 2 (*hETV2*) in hESCs, Cakir et al. (2019) generated cortical organoids, termed vascularized human cortical organoids, with a complex vascular network and more mature neurons. Vascularized human cortical organoids display a vascular-like phenotype, express endothelial and blood–brain barrier markers, and contain tight junctions similar to those observed in 3D blood–brain barrier models. Their results showed that incorporating 20% of these cells was optimal for organoid construction, highlighting the significance of detailed considerations and technical refinements when simulating the physiological states of hman brain.

### Cracking the genetic code of human brain evolution and development

The complexity of the human nervous system, neurodevelopment, and neurological diseases is unparalleled. Understanding human brain evolution is crucial for advancing research on neurodevelopment and neurological diseases. Identifying key genes directing human brain evolution and development, as well as deciphering their regulatory networks, are major goals in the field. Human brain organoid models preserve gene regulatory networks linked to primary cell types and developmental processes (Pollen et al., 2019). Moreover, they can markedly reduce technical challenges, as well as times and costs required for research on human brain evolution and development. Based on genomic data and relevant literature, candidate genes can be identified and knocked out using genome editing technology in human PSCs and/or brain organoids to investigate their neural developmental function (Qian et al., 2022). For example, neuro-oncological ventral antigen 1 (*NOVA1*) encodes RNA-binding splicing regulator, an evolutionarily conserved neuro-oncological ventral antigen, and assumes key roles in brain development and tumorigenesis (Trujillo et al., 2021). CRISPR/Cas9-mediated genome editing technology was employed to replace the modern human *NOVA1* gene with Neanderthal and Denisovan variants in cortical organoids. This genetic modification was found to retard neural development and result in higher surface complexity and altered electrophysiological properties in cortical organoids expressing the archaic *NOVA1* variant. Transketolase like 1 (*TKTL1*) is another gene involved in cortical development in modern humans (Pinson et al., 2022). To investigate differences between modern human *TKTL1* (*hTKTL1*) and Neanderthal *TKTL1* (*aTKTL1*), *aTKTL1*-hESC line was created by CRISPR/Cas9 editing and induced to form brain organoids. The *aTKTL1*-expressing organoids radiated fewer oRGs and neurons than *hTKTL1*-expressing organoids. CRISPR/Cas9-mediated genome editing in brain organoids also helped to establish the role of human-specific notch 2 N-terminal like (*NOTCH2NL*) locus in cortical expansion and size control (Fiddes et al., 2018). The downregulation of neuronal differentiation by human-specific *NOTCH2NL* expression resulted in an overall final increase in neuronal production. A recent study investigated the genetic basis for the larger and more complex human forebrain compared to that of chimpanzees, revealing that the transcription factor zinc finger protein 558 (ZNF558) is expressed exclusively in human forebrain NPCs (Johansson et al., 2022). Subsequently, the researchers examined the role of ZNF558 in brain organoids using CRISPRi technology. They found that silencing ZNF558 did not affect organoid production; however, it led to the production of smaller organoids at earlier stages of differentiation and more mature neurons at later stages.

### Lineage tracing and high-throughput screening

Lineage tracing is a fundamental technique in developmental biology (Qi et al., 2025). As early as the 19^th^ century, researchers used visual lineage tracing with peroxide or protein antibody markers to trace the developmental origins of neural cells (Kretzschmar and Watt, 2012). With the rapid progress in high-throughput sequencing technologies and CRISPR/Cas9 genome editing technology, genetic lineage tracing has advanced and precipitated our understanding of neural development and disease tremendously (VanHorn and Morris, 2021). Previous reviews have discussed in detail the history, principles, and construction strategies of lineage tracking (Wagner and Klein, 2020; VanHorn and Morris, 2021). This review primarily focuses on introducing the potential application of relevant lineage tracking strategies in brain organoids.

Among the numerous lineage tracing techniques, gene barcoding has emerged as the most powerful and broadly adapted approach. Gene barcodes suitable for cell tagging and lineage tracing are inheritable genetic markers either generated based on molecular genetics or artificially created by gene engineering, such as CRISPR/Cas9-mediated genome editing in interested cells (Chen et al., 2022; Xu et al., 2024). CRISPR/Cas9 barcoding enables the tracing of a cell’s developmental history, which allows the tracking of millions of cells in parallel. When combined with single-cell transcriptomics, this technique is capable of analyzing cell types and constructing cell lineage maps at various developmental stages, thereby facilitating the study of heterogeneous cell populations and their lineages (Kebschull and Zador, 2018). Over the years, research groups have investigated various strategies. McKenna et al. (2016) designed a Genome editing of synthetic target arrays for lineage tracing (GESTALT) system based on CRISPR/Cas9 technology enabling progressive introduction and accumulation of mutations in the DNA barcodes over multiple rounds of cell division in zebrafish. Subsequently, the GESTALT system was improved through combination with single-cell sequencing technology to construct single-cell RNA sequencing synthetic target arrays for lineage tracing (scGESTALT) and effectively enables tracking the development of single cells rather than clones (Raj et al., 2018). Spanjaard et al. (2018) reported the LINNAEUS (lineage tracing by nuclease-activated editing of ubiquitous sequence) system, which uses CRISPR/Cas9 to cleave a red fluorescent protein (*RFP*) transgene, prompting the cell to repair its DNA and create a genetic scar that serves as a recognition marker. Weinreb et al. (2020) developed a LARRY (Lineage And RNA RecoverY) system to deliver DNA barcodes by lentiviral delivery. These gene barcode technologies have significantly expanded the toolkit for lineage tracing and have been rapidly applied in the field of neurobiology and organoid models.

Due to the limited accessibility of human brain tissue, lineage tracing traditionally relies on mouse and cell culture systems. While differences in neural development between humans and rodents limit the relevance of many studies to human biology, brain organoids offer a promising experimental approach. He et al. (2022) developed iTracer, a lineage recorder that combines reporter gene barcoding with inducible CRISPR/Cas9 scar formation, compatible with single-cell and spatial transcriptomics. They discovered that neural fate determination in brain organoids occurs early, between the progenitor cell stage and neuroectoderm formation. Their further functional and mechanistic exploration of TSC complex subunit 2 (*TSC2*) gene demonstrated the utilities of this system for disease research and molecular studies. You et al. (2023) developed a single-cell split barcoding system, named SISBAR, based on the idea that newly dividing sister cells can serve as surrogates for each other because they are transcriptionally similar and have the same fate. With this strategy, single-cell RNA sequencing and viral barcoding retrieval were performed twice at the early and late differentiation stages. Subsequently, lineage trees could be established through computational comparison and reconstruction between the timepoints. The Chen group has also developed a CRISPR editing-based lineage-specific tracing (CREST) method that combines recombinase system and single-cell sequencing technology, and enables single-cell spatiotemporal lineage mapping of mouse mesencephalon embryonic development (Xie et al., 2023). Fleck et al. (2023) developed a multimodal omics technique—including single-cell sequencing, chromatin accessibility, and genetic perturbation—combined with brain organoid technology to investigate DV telencephalon differentiation. They infected an inducible Cas9 cassette containing hiPSCs with a lentiviral library of guide RNAs targeting 20 transcription factors and screened for transcription factors in human brain development with organoid models. This strategy enabled semi-high-throughput screening of molecules crucial for human neural development. The investigators found that transcription factor glioma-associated oncogene family zinc finger 3 (GLI3) was required to establish cortical fate. This high-throughput strategy has the potential to predict transcriptome regulatory networks at genome-wide level. Furthermore, when combined with organoid models, it provides a new systematic approach to dissecting human neural development.

Lineage tracing technology combined with brain organoid models has also shown great power in studying neurological diseases. Increasing evidence suggests the existence of multiple gene regulatory networks in ASD (He et al., 2022). Li et al. (2023) developed and utilized the CRISPR–Human Organoids–Single-Cell RNA sequencing (CHOOSE) system to screen vulnerable cell types and gene regulatory networks linked to ASD based on single-cell transcriptome and chromatin patterns. The CHOOSE system enables functionally targeting and dissecting one specific gene mutation per cell in a high-throughput manner. With successful application of the CHOOSE system to brain organoids, the researchers tracked at the single-cell level each ASD-associated gene mutation and mapped the developmental trajectories of the targeted cells. Through this approach, they uncovered the effects of mutations in 36 autism risk genes on the transcriptional regulation of neural cell fates.

Since lineage tracing can trace the progenies of any given progenitor cell, it is also a powerful tool for studying tumors, particularly those with high heterogeneity (e.g., gliomas). In an earlier study, Mohme et al. (2017) developed a CRISPR-independent fluorescent ‘optical barcoding’ technique to track two glioma cell lines. The incorporation of fluorescent proteins enabled rapid and sensitive tracking of clonal properties with flow cytometry. They used a binary/digital flow cytometry-based readout to encode colors and distinguish 41 clones. However, the limited encoding capacity of such optical barcodes is prohibitive in capturing all the heterogeneity within tumors. To capture and dissect rare, transiently resistant, and persistent proliferative cancer cell subpopulations, Oren et al. (2021) developed Watermelon, a sophisticated lentiviral barcoding library containing > 5 million genetic codes. This library enables the tracing of a cell’s clonal lineage and gene expression, permitting the identification of rare persister lineages and dissection of their molecular states. They demonstrated that cycling and non-cycling persisters arose from different cell lineages of distinct transcriptional and metabolic programs and analyzed cells preferentially poised to proliferate under treatment with drugs. Watermelon primarily targets drug-resistant cancer cells, though its application in this study was limited to 2D cell models. Given the potential for efficiency changes in 3D systems, the development of CRISPR-based techniques for investigating brain tumors is highly desirable.

### Disease modeling

Brain organoids derived from gene-edited cell lines are increasingly serving as effective models for studying neural developmental disorders, enabling exploration of the interactions between disease phenotypes, genetic variants, and gene expression. Disease modeling in brain organoids primarily depends on PSCs. Pathogenic mutations can be introduced into healthy cells via gene editing (knockin/knockout) and subsequently induced to form brain organoids with disease-related genes. This approach plays a critical role in studying neurogenetic diseases caused by single-gene mutations (Xiang et al., 2020). Microcephaly, a common NDD, involves various pathogenic mechanisms. CRISPR/Cas9 mediated occludin-specific (*OCLN*-specific) deletion in brain organoid development resulted in organoids size deficit, premature cell cycle exit, and early neuronal differentiation, consistent with microcephaly and polymicrogyria observed in patients (Bendriem et al., 2019). WD repeat domain 62 (*WDR62*) knockout resulted in a reduction of oRG cells, slower NPC proliferation, and premature entry into differentiation (Zhang et al., 2019). For complex genetic diseases, brain organoids can be derived from iPSCs. Brain organoids derived from iPSCs of patients with Down syndrome (DS) showed clinically relevant phenotypes and CRISPR/Cas9-mediated knockdown of the aberrant DS cell adhesion molecule (*DSCAM*) expression or correction of the dual specificity tyrosine phosphorylation regulated kinase 1A (*DYRK1A*) gene copy number could cellularly and molecularly abolish the diseased phenotypes (e.g., proliferation deficit, aberrant neurogenesis and accelerated aging) (Tang et al., 2021; Xu et al., 2022; Murray et al., 2023). Chromodomain helicase DNA-binding protein 8 (*CHD8*) is a frequently mutated gene associated with ASD, and *CHD8* knockout cell lines have been extensively studied using monolayer cell culture models (Wang et al., 2015; Shi et al., 2023). Wang et al. (2017) compared brain organoids to NPCs and monolayer neurons and reported that nearly 50% of differentially expressed genes in organoids showed differential expression in NPCs and monolayer neurons. Beyond single-gene studies of NDDs, a recent study presented a noteworthy approach. To investigate the role of 425 NDD-related genes in neurodevelopment, researchers constructed single-guide RNA libraries to infect hiPSCs (Meng et al., 2023). Subsequently, they induced the formation of human subpallial organoids and assembled them with human cortical organoids, identifying genes potentially involved in the migration of interneurons. This evidence highlights the powerful combination of CRISPR screening and brain organoids in uncovering gene functions.

Genome editing of brain organoids also offers a novel approach to simulating brain tumors associated with high mortality rates for which a limited number of research models exist. Brain tumorigenic mutations can be easily introduced through genome editing into brain organoids to create 3D brain tumor models *in vitro*, thus enabling mechanistic and drug screening studies. Gliomas, the most common malignant brain tumors, are often driven by mutations in specific genes. Kim et al. (2021) used CRISPR/Cas9 technology to introduce the epidermal growth factor receptor variant III (*EGFRvIII*) mutation into hESCs and showed that *EGFRvIII* mutation induced temozolomide-responsive astrogenesis and marked cell proliferation in brain organoids. Patched 1 (*PTCH1*) is a key receptor in the SHH pathway and loss-of-function mutations in *PTCH1* are associated with medulloblastoma. van Essen et al. (2024) introduced *PTCH1* mutations and disrupted cerebellar organoid development via the SHH signaling pathway, resulting in features associated with pre-neoplastic stages of medulloblastoma.

Somatic cells from patients can also be reprogrammed into iPSCs, genetically modified to correct mutations, and utilized for functional validation. The loss of fragile X mental retardation protein (FMRP) causes fragile X syndrome. Kang et al. (2021) created both fragile X syndrome patient-specific and gene-corrected iPSCs lines and induced them to form forebrain organoids. They confirmed that loss of the protein impaired NPC proliferation and neuronal differentiation and enhanced synapse formation. RNA sequencing analysis revealed significant differences in mRNA expression and various pathways between fragile X syndrome forebrain organoids and gene-corrected controls. Among them, inhibiting the phosphoinositide 3-kinase (PI3K) pathway could reverse symptoms, and chromodomain helicase DNA binding protein 2 (*CHD2*) was identified as a major human-specific mRNA target of FMRP. CRISPR-based single-base editors offer a precise and efficient method for repairing single bases in DNA or RNA in diseases caused by point mutations (Fu et al., 2023). This technique allows for the generation of isogenic iPSC lines that do not carry mutations, providing valuable control within the patient’s genetic background. Nonetheless, scientists must address the limitations of brain organoids representing embryonic, rather than adult, stages. In future research, accelerating development and aging in 3D organoids *in vitro* may be necessary to study degenerative diseases. Researchers are also exploring more efficient and convenient methods to increase the efficiency and flexibility of genome editing in organoids. For example, two studies created gene knockin and knockout in human liver ductal organoids by injecting plasmids into organoids with basement membrane extract, a technique similar to uterine electroporation, named CRISPR-HOT (Artegiani et al., 2020; Hendriks et al., 2021). This technique is based on non-homologous end joining-mediated introduction of indels into the endogenous loci of organoids rather than homology-directed repair. Positive organoids were selected based on plasmids constitutively expressing a fluorescent reporter. In the future, a similar approach could be applied to brain organoids for development and disease modeling.

## Conclusion

In this review, we provide an overview of the background and historical development of brain organoids, classify the different types of brain organoids, and explore their application in neurodevelopment and related diseases. In addition, after briefly discussing genome editing technologies, we focus on summarizing the intersection of genome editing techniques with brain organoid research.

Science is inherently interdisciplinary, and the integration of diverse technologies often leads to different outcomes. Many contemporary brain organoid studies have incorporated innovations from materials science. One such development is the replacement of complex Matrigel with hydrogel as an extracellular matrix, which enhances the controllability of the entire culture process (Kjar et al., 2024). Another approach supporting brain organoid culture involves the addition of fibers to the organoid structure (Zhu et al., 2017; Tejchman et al., 2020; Giandomenico et al., 2021). The ultimate goal of these attempts is to address a major challenge in brain organoid research, namely variability. This challenge, often resulting from human intervention, remains a significant obstacle. Reducing manual manipulation is essential, and engineered culture processes—such as molds for forming aggregates—show great promise in producing more consistent samples and enabling large-scale, high-throughput experiments (Qian et al., 2016). Furthermore, 3D printing technology, when combined with biological materials, can be standardized to produce aggregates, thereby enhancing the consistency of brain organoids (Cadena et al., 2024). Microfluidic technology offers a more stable culture environment, enhances gas exchange, mitigates organoid necrosis due to hypoxia, and enables greater control over the cultivation conditions (Cho et al., 2021; Lokai et al., 2023).

Another significant challenge in brain organoids is the lack of cells from other lineages. To address this, researchers have primarily focused on co-culturing these cells or organoids, which requires optimization of the culture medium to maintain optimal growth of all cell types. Alternatively, transplanting brain organoids into animal models, such as mouse brains, enables the infiltration of microglia and blood vessels (Schafer et al., 2023). However, this approach increases experimental complexity and is less suitable for large-scale drug screening. Researchers have attempted to reduce ectodermal differentiation and promote the self-generation of microglia by modulating neural media composition (Ormel et al., 2018). While this strategy shows promise, it compromises neural development and leads to an uncontrollable microglial population. Genome editing technologies offer a potential solution by enabling the ectopic expression of genes to introduce non-ectodermal cells into the brain organoid (Cakir et al., 2022). The future integration of genome editing could allow for better control over the number and types of non-ectodermal cells within the organoid to more closely resemble a physiological state.

The process of brain organoid culture is lengthy, often lasting several months, and the maturation is slow. For instance, the generation of glial cells occurs late in development, making it challenging to use immature brain organoids to study adult-onset neurological diseases, such as AD. The extended culture time presents significant challenges for researchers, increasing experimental costs and slowing the overall pace of research. However, gene editing technologies offer the potential to accelerate this process by promoting the expression of neurodevelopmental genes, such as neuronal differentiation 1 (*NeuroD1*), thereby enhancing organoid maturation (Choi et al., 2020; Zhao et al., 2021; Ciceri et al., 2024). Additionally, the expression of aging-related genes could be used to artificially induce aging in brain organoids, thereby expanding the scope of research into age-related neurological diseases (Miller et al., 2013).

Genome editing technologies have the potential to significantly advance the study of neurodevelopment and neurological diseases using brain organoids. Gene editing can be employed to investigate the role of individual genes in these processes. A key aspect of such studies is examining dose-dependent gene effects, which can be achieved by inserting specific elements that regulate the expression levels of target genes (Zhu et al., 2019). For genes that function at different stages of development, the introduction of regulatory sequences to establish gene ‘switches’ allows for the controlled activation or repression of gene expression at specific time points, enabling the study of temporal gene function. Although technologies such as PE and BE, which are derived from CRISPR/Cas9, have yet to be widely applied in brain organoids, their future use in this context holds considerable promise. For the study of genetic diseases caused by base mutations, BE and PE can accurately complete correction or mutation, thereby reducing the risk of CRISPR/Cas9 off-target effect. Another promising area of research is the integration of brain organoids with CRISPRi and CRISPRa systems for large-scale genetic screening. This approach enables researchers to rapidly identify genes that regulate neuronal function, while also facilitating the study of complex neural regulatory networks. CRISPR screens have already been employed for gene screening in NDDs, and this approach may reveal gene regulatory networks underlying other neurological disorders in the future (Meng et al., 2023).

The advent of brain organoids has revolutionized the methods and tools to approach outstanding questions related to human-specific brain development and diseases, while the combination of genome editing and organoid technologies offers great promise. Many challenges remain in this field. Thus, inventing and optimizing induction protocols with better structural representation, more diversity, and higher cellular complexity and maturity shall be a key research focus in the near future. New organoid engineering and genome editing approaches should also be pursued and continuously optimized to facilitate accessibility, flexibility, and scalability. This represents a significant advancement in studying human neural genetics in a 3D environment that more closely mimics the physiological context, driving progress in the field. As biology enters a big data era, brain organoids empowered by fast evolving genome editing technology hold unprecedented promises in both basic research and clinical applications.

Although an extensive search was conducted, a comprehensive synthesis of all relevant studies was not feasible, leading to inherent limitations in this review. Firstly, this article focuses on two primary topics, namely brain organoids and the integration of gene editing technologies with brain organoids. Hence, it excludes the application of other technologies within the realm of brain organoid research. As a result, the scope of this review is inherently limited, with no comprehensive exploration of potential interdisciplinary integrations, such as materials science and 3D printing technologies that could complement brain organoid research. Secondly, this review primarily emphasizes the application of brain organoids and gene editing technologies in neurodevelopment and neurological diseases, with limited discussion of the underlying molecular mechanisms and signaling pathways. This focus may lead to an incomplete understanding of the biological mechanisms driving neurodevelopment and disease processes. Finally, the selective nature of published studies, particularly those reporting positive outcomes, may introduce a bias, thereby potentially skewing the overall representation and balance of this review. For beginners, it is important to be aware of these limitations and to read this review and navigate the field in a broader context.

## Additional files:

***Additional Table 1:*** *Summary of unguided brain organoid protocols.*

***Additional Table 2:*** *Summary of guided brain organoid protocols.*

***Additional Table 3:*** *Schematic overview of brain assembloids.*

***Additional Table 4:*** *Summary of commonly used genome editing and related gene expression manipulation techniques.*

## Data Availability

*All relevant data are within the paper and its Additional files*.
